# Understanding the HIV coreceptor switch from a dynamical perspective

**DOI:** 10.1186/1471-2148-9-274

**Published:** 2009-11-30

**Authors:** Christel Kamp

**Affiliations:** 1 Paul-Ehrlich-Institut, Paul-Ehrlich-Straße 51-59, 63225 Langen, Germany

## Abstract

**Background:**

The entry of HIV into its target cells is facilitated by the prior binding to the cell surface molecule CD4 and a secondary coreceptor, mostly the chemokine receptors CCR5 or CXCR4. In early infection CCR5-using viruses (R5 viruses) are mostly dominant while a receptor switch towards CXCR4 occurs in about 50% of the infected individuals (X4 viruses) which is associated with a progression of the disease. There are many hypotheses regarding the underlying dynamics without yet a conclusive understanding.

**Results:**

While it is difficult to isolate key factors *in vivo *we have developed a minimal *in silico *model based on the approaches of Nowak and May to investigate the conditions under which the receptor switch occurs. The model allows to investigate the evolution of viral strains within a probabilistic framework along the three stages of disease from primary and latent infection to the onset of AIDS with a a sudden increase in viral load which goes along with the impairment of the immune response. The model is specifically applied to investigate the evolution of the viral quasispecies in terms of R5 and X4 viruses which directly translates into the composition of viral load and consequently the question of the coreceptor switch.

**Conclusion:**

The model can explain the coreceptor switch as a result of a dynamical change in the underlying environmental conditions in the host. The emergence of X4 strains does not necessarily result in the dominance of X4 viruses in viral load which is more likely to occur in the model after some time of chronic infection. A better understanding of the conditions leading to the coreceptor switch is especially of interest as CCR5 blockers have recently been licensed as drugs which suppress R5 viruses but do not seem to necessarily induce a coreceptor switch.

## Background

Although many studies aim to understand the evolution of the human immunodeficiency virus (HIV) in its host [1-3] there are still a lot of open questions related to the mechanisms driving the intra-host dynamics. One of these puzzles is associated with the HIV coreceptor switch [4]: To facilitate cell entry and subsequent replication HIV binds to the cell surface molecule CD4 as well as a chemokine coreceptor, most commonly the CCR5 or CXCR4 coreceptors. In most patients viruses that use the CCR5 coreceptor (R5 viruses) dominate in early stages of disease. This preference changes however in about 50% of patients during the course of disease towards viruses using the CXCR4 coreceptor (X4 viruses). This switch in coreceptor usage is associated with a worsened prognosis which makes a better understanding of the switch dynamics of direct clinical importance. At the same time there are several explanatory hypotheses but not yet a conclusive understanding of the underlying processes in terms of empirical evidence or model support. The transmission mutation hypothesis [4] assumes that R5 viruses are favoured in transmission and are in consequence more often found in early infection. X4 viruses with higher fitness can emerge from R5 viruses via intermediate mutants of lower fitness which will finally become dominant [4]. While there is some evidence for a fitness loss of intermediate mutants [5-7], this hypothesis strongly relies on the assumption that X4 viruses can hardly be transmitted in infectious doses as these would otherwise immediately become dominant due to their assumed selectional advantages in the host. There might be some strain selection during transmission but there is evidence that both R5 and X4 strains can be transmitted in infectious doses, however, with X4 viruses seemingly having a selectional disadvantage thereafter [8,9]. The effective replication rate of X4 viruses exceeds R5 replication in some *in vitro *assays [10], there is, however, no conclusive picture of the *in vivo *situation with different environmental constraints [4,11]. Taken this together [4,12-14], it is highly questionable whether a fragile setting relying solely on the transmission mutation hypothesis adequately describes the major processes in place or robustly represents the observed dynamics.

An alternative and less fragile hypothesis assumes that environmental conditions in the host change during the course of disease in favour of X4 viruses. Such changes may be the consequence of immune pressure or the availability of adequate target cells for replication in terms of (co-)receptor expression or replication efficiency by stage of cell development (naive/memory cells) [4,15]. In a recent study Sguanci and coworkers [16] investigate the changing environment by assuming that the susceptibility of target cells (or infectivity of viruses) increases in favour of X4 cells as soon as these emerge in an auto-catalytic fashion, however, staying with the assumption that the initial infection is with R5 virus only. Earlier work by Wodarz et al. [17] and Callaway et al. [18] allows more realistically for a mixed infection and investigates how the shift in target cells and HIV specific T cells can dynamically induce a shift from R5 to X4 viruses. In a similar fashion, Ribeiro et al. investigate how the coreceptor switch can be facilitated due to preferential replication of X4 and R5 viruses in naive and memory T cells, respectively, with differences in efficiency of viral reproduction [15]. These approaches support the hypothesis of environmental change as a driving force to the coreceptor switch but do not consider the stochastic nature of virus evolution and the emergence of new strains and viral diversity which have been associated with the coreceptor switch. While the relative importance of the above hypotheses for the coreceptor switch can ultimately only be answered experimentally we will here focus on the more robust hypothesis of environmental change for further analysis. We want to identify key processes resulting in the observed phenomenon by a restriction to a minimal model representing the dynamics of the immune system and HIV [19]. Therefore, we develop an approach based on the well studied model of Nowak and May [20] that allows to study the dynamical processes associated with the HIV coreceptor switch in combination with viral evolution. While we do not go yet into the details of cellular subpopulations whose roles are empirically still under debate we rather take advantage of the relative simplicity of the model to study the effects of the stochastic nature of HIV evolution neglected in the models outlined above. The stochastic framework will allow to analyse the distribution of evolutionary pathways associated with the coreceptor switch as well as survival times in a more systematic way.

It has specifically become of major importance to understand the processes leading to the dominance of X4 viruses since new drugs have been licensed that block the CCR5 receptor [12,21-23] and in consequence can shift the selective advantage towards X4 viruses [24], although not necessarily inducing a coreceptor switch. Considering the association between X4 viruses and a worsened prognosis [4,25] it is of vital importance to understand whether X4 can only flourish in the environment of a patient weakened by side effects of a chronic infection or whether X4 viruses themselves destabilise the patient and cause the worsened prognosis [13].

## Results and Discussion

### The Model

Following the ansatz of Nowak and May [20,26] we study the time evolution of viral load *v*_*i *_of a set of *n *strains (*i *∈ {1, ...,*n*}). These are controlled by a specific immune response with strength *x*_*i *_and as well as a cross-reactive immune response with strength *z*. Their time evolution is determined by the following set of equations:

with variables and parameters as summarised in table 1.

**Table 1:** Variables and parameters in equations (1)

*v*_*i*_	viral load from strain *i*
*x*_*i*_	specific immune response to strain *i*

*z*	cross reactive immune response

*r*_*i*_	growth rate of virus *i*

*p*_*i*_, *q*_*i*_	effect of specific and cross reactive immune response on strain *i*

*c*_*i*_, *k*_*i*_	growth of specific and cross reactive immune response induced by and directed against strain *i*

*b*	natural decay of the immune response

*u*_*i*_	decay of immune response induced by strain *i*.

The viral load of strain *i *grows at a rate *r*_*i *_and is diminished by the specific and cross-reactive immune response at a rate -*p*_*i*_*x*_*i *_- *q*_*i*_*z*. Vice versa, a specific immune response is stimulated by each strain as is the cross-reactive immunity by the total viral load. Immune responses decay at a rate *b *and further at a rate proportional to the viral load which is to account for the fact that HIV infected T helper cells are depleted (and in consequence impair the immune response mediated by other T and B cells). Among the 2^*n *^possible equilibrium solutions of a set of equations (1) for *n *strains at most one is stable [20], that is, for any number of strains the viral load either diverges or converges to a well defined equilibrium viral load. Strains are distinguished by their sensitivity towards the specific immune response, i.e. several related genomic sequences might represent the same epitope and consequently correspond to one strain in the model. The model does not yet address the question of different immune cell populations as for example CD4 vs. CD8 cells, naive vs. memory cells or resting vs. replicating cells. The distinction between *x*_*i *_and *z *takes only into account that there is a strain specific and an unspecific component of the immune response. The latter can be a composition of the response towards conserved parts of the virus, of cross-reactivity from earlier specific immune responses or innate immunity. Bare of any strain-specific responsiveness, *z *is a measure for the general activation of the immune system going along with an overall turnover of CD4 cells (irrespective of their specificity) seen in HIV infections [27,28].

To focus on the evolution of R5 and X4 viruses in a patient we distinguish only between two parameter sets corresponding to either type of virus as listed in table 2. The values of initial viral growth rates are estimated from [29,30]. However, viral growth rates or more generally viral fitness as the balance between viral replication and decay is not an intrinsic feature of the virus but is only well defined in the context of the viruses environmental conditions. Differences in viral fitness may therefore arise among viruses and over time with a change of environmental conditions such as the availability of target cells or the strength of an immune response. Here, we assume that X4 viruses are better recognised by the specific immune response than R5 viruses [31] and increase their replication rate proportional to the stimulation and activation of the immune system [15,27,28] represented by the cross-reactive immunity *z*. While the cumulative immune activation *z *will likely impact on both R5 and X4 viral replication [15,32] we are only interested in the increase of X4 replication over R5 replication and therefore keep the R5 replication rate fixed. The cumulative immune activation *z *increases slightly during the course of disease and breaks only down with the collapse of the immune system at the onset of AIDS. The chosen parameter set corresponds to a situation in which the viral load can initially be controlled by the immune response, i.e. a situation in which limitations in target cell supplies or other saturation phenomena do not have to be taken into account. The model's dynamics is robust with respect to the choice of further parameters as long as they correspond to the model's regime of HIV-like dynamics (cf. equation (4)).

**Table 2:** Parameter settings

parameter	R5 virus	X4 virus
number of strains	*n*^*R*5^	*n*^*X*4^

viral growth rate *r*_*i*_	*r* (2*d*^-1^)	*r*(1 + *αz*) ((2 + *z*)*d*^-1^)

effect of specific immune response *p*_*i*_	*p*^*R*5^ (2*d*^-1^)	*p*^*X*4 ^>*p*^*R*5^ (20*d*^-1^)

effect of cross-reactive immunity *q*_*i*_	*q* (1.86*d*^-1^)	

stimulation of specific and cross-reactive immunity *c*_*i*_, *k*_*i*_	*c*, *k* (0.1*d*^-1^*V L*^-1^)	

natural and virus induced depletion of immune response *b*, *u*_*i*_	*b*, *u* (0.02*d*^-1^, 0.1*d*^-1^*V L*^-1^)	

probability to generate a new strain *p*_*m *_per day and viral load	(0.005*d*^-1^*V L*^-1^)	

Parameter settings for R5 and X4 viruses, the values chosen in the simulations are given in brackets in units per day (d) and viral load (VL), due to the close genetic neighbourhood of R5 and X4 viruses it is assumed that both mutants occur with the same probability  from the total viral population irrespective of its composition. Where direct biological interpretation is possible parameters have been chosen following [29,30], remaining degrees of freedom have been fixed to account for the appropriate dynamical regime defined by equation (4). Note that the growth rate of *X*4 viruses is assumed to increase proportional to the immune activation measured by the cross reactive immunity *z *with *α *= 0.5.

The model system is initialised with a strain of R5 virus and a strain of X4 virus and evolves according to equations (1). A new mutant arises at a hazard rate *p*_*m*_*v*, i.e. at each integration step of length *dt *with probability . In the current setting, X4 and R5 mutants are assumed to arise with equal probability irrespective of the composition of the viral load due to the close genetic neighbourhood of these viral subtypes [33,34] (for further discussion cf. Additional file 1, Figure S1). After the emergence of each new mutant the system reaches a new equilibrium with *n*^*R*5 ^R5 virus strains and *n*^*X*4 ^X4 virus strains and viral load

The system is stable, i.e. there is a stable equilibrium solution only if

1. *ru *<*kq*, i.e. the viral load is fully controlled by the cross reactive immune response *z *alone or

2.

that is, only as long as the numbers of R5 and X4 virus strains do not exceed the critical numbers given by condition (3) the immune system can control the infection.

We are specifically interested in dynamical regimes of the model which remain only stable for finite numbers of *R*5 and *X*4 virus strains (as opposed to case 1.) but do not show immediate divergence (*cp*^*R*5 ^- (*ru *- *kq*) > 0) which corresponds to

The first inequality in (4) implies that the cross-reactive immune response cannot fully control the viral infection as can be seen from equation (2). This means that each viral strain needs to be suppressed both by a specific and a cross-reactive immune response. The second inequality implies that the immune system can control only a limited number of strains, i.e. the limitations of the cross-reactive immune response can be compensated by the specific immune response if the critical number of strains given by equation (3) is not exceeded. As soon as this number of strains is exceeded this will lead to an uncontrolled increase in viral load in the model corresponding to the onset of AIDS in the patient [20]. The detailed dynamics in this regime are not longer described by equations (1) as saturation effects have to be considered due to limited resources.

The emergence of viral strains in a patient that accumulate to the critical number at the onset of AIDS can also be understood in a probabilistic framework in order to study survival distributions. Therefore we derive the probability *P*(*n*^*R*5^, *n*^*X*4^, *t*) that a patient harbours *n*^*R*5 ^strains of *R*5 virus and *n*^*X*4 ^strains of *X*4 virus at time *t*. If the viral load equilibrates much faster after the emergence of a new mutant than the next mutant arises we can assume that new mutants will approximately arise at a rate proportional to the equilibrium viral load *v*(*n*^*R*5^, *n*^*X*4^). In the regime where  and *v*(*n*^*R*5^, *n*^*X*4^) <*v*_*max *_the evolution of the number of viral strains is then described by a Markov process with the master equation [35]

For those states in which the critical number of strains given by equation (3) or a maximal viral load *v*_*max *_is exceeded, i.e. at the onset of AIDS, the last term in equation (5) is defined to vanish making these states absorbing states of the Markov process. *v*_*max *_limits the diverging viral load *v*(*n*^*R*5^, *n*^*X*4^) as the total viral load in the patient is limited. The distribution of times until absorption, i.e. the survival distributions, are described by phase type distributions [36].

### The course of disease

**Figure 1:**
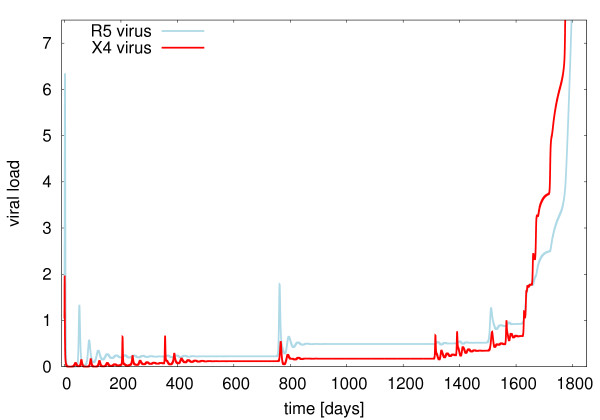
**The course of disease in the model**. Viral load of R5 viruses (blue) and X4 viruses (red) in a simulation of the model described by equations (1) and parameters as in table 2. The viral load is given in arbitrary units of viral load (VL).

First insight into the evolutionary dynamics of R5 and X4 viruses can be gained by investigating the time course of viral load attributed to each subtype in the model. Such a typical course of disease after a mixed infection with R5 and X4 viruses in the model is shown in Fig. 1.

The system shows a period of low viral load after the initial phase of disease with is interspersed with small outbreaks when a new viral mutant occurs - until eventually the viral load rises again. The system shows a quasi-stable behaviour as long as inequality (3) can be fulfilled for a non-zero but limited number of strains, i.e. if inequality (4) holds.

X4 viruses are underrepresented in viral load in the early stages of disease as the model assumes that they are more strongly suppressed by the specific immune response than R5 viruses (*p*^*X*4 ^> *p*^*R*5^) [31] while having initially identical growth rates [11]. On the other hand the X4 replication rate in the model increases proportional to the overall activation of the immune system represented by the cross-reactive immune response. This shifts fitness advantages from R5 viruses to X4 viruses resulting in the phenomenology of a coreceptor switch.

The model captures the association between ongoing immune activation and the apparent shift in evolutionary advantages from R5 to X4 viruses during an HIV infection. There is however still controversy about the mechanisms underlying this association. Earlier assumptions about a shift in target cells favouring X4 over R5 viruses [4] are now questioned [37]. An alternative hypothesis links the efficiency of viral production to the division rate of target cells [15]. With the differential increase in target cell division rates during chronic infection [32] viral replication rates are differentially increased which eventually shifts the selectional advantage from R5 viruses to X4 viruses [15,27,28]. Another interpretation of the model equations is that X4 viruses experience a stronger specific immune response than R5 viruses but that they face a reduced cross-reactive immune response. In consequence X4 viruses will be more difficult to control as the number of strains grows. Any of these hypotheses allows for a dynamical change in environmental conditions in the host that become more favourable for X4 viruses after chronic infection while selection is in favour of R5 viruses in early stages of disease. Although, there is some evidence [4,11,15,31,32] in accordance with the model's assumptions on parameter settings it is difficult to get conclusive results from currently available empirical data. Many studies with one or only a few HIV or SIV cases point in different directions [5-7	,38]: Any comparative measurement of growth rates and the effect of an immune response among strains *in vivo *assesses quantities derived from a large variety of underlying processes that might vary among cases. Therefore, a more conclusive picture can only be expected with the integration of more data describing the interactions between HIV and the immune system in many patients. The observation that some viral strains may be present in a patient for a long time before they appear in detectable numbers favours the idea of a dynamical change in the environmental conditions in favour of X4 viruses [39,40] at a higher level of abstraction. Reflecting these findings, the current model provides a coarse grained picture of possible scenarios for environmental shifts in the patient for further investigation. While details of these dynamics will have to be addressed within a refined model the current model has the advantage to be accessible to further analysis of the basic underlying stochastic processes as demonstrated in the following sections.

### The routes of evolution

The probabilistic framework complementarily allows to study the probability to find a setting with *n*^*R*5 ^R5 strains and *n*^*X*4 ^X4 strains in a patient at any time during the course of disease - including the probability to have reached the final stage of disease, i.e. having exceeded the critical number of strains.

**Figure 2:**
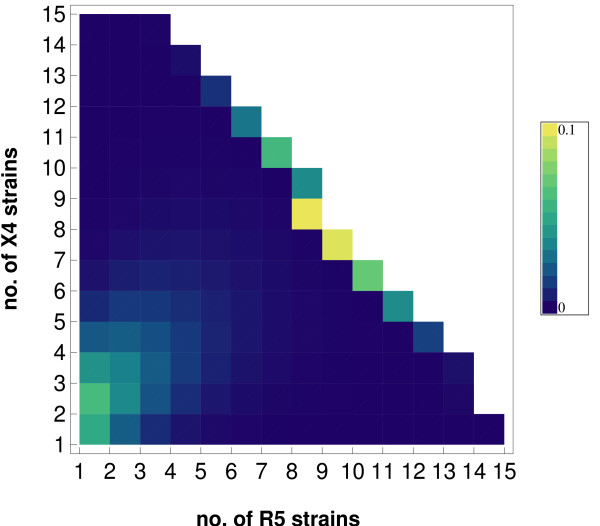
**The distribution of strains**. The probability to find *n*^*R*5 ^R5 strains and *n*^*X*4 ^X4 strains in a patient at 1200 days since infection in the model with parameters as in table 2 and *v*_*max *_= 500 units of viral load, the final stage of disease (absorbing state) is marked by the accumulation of probability density at the diagonal border.

Fig. 2 shows that a patient is most likely to be found either with a few strains or in a situation in which the final stage of disease has already been reached. This is due to the fact that the viral load and in consequence the probability to generate new mutants increases with a growing burden of viral strains. As a result the mean residence time with a given number of strains  will decrease as the number of strains grows, i.e. the course of disease speeds up with the accumulation of viral strains. The distribution of strain compositions shown in Fig. 2 is asymmetric with respect to the numbers of R5 and X4 strains in the presence of a moderate number of strains, i.e. in a stage of disease that is not yet progressed. A moderate number of strains will not result in strong stimulation of the immune system and X4 replication and viral load remain at a low level compared to the contribution of R5 strains. X4 strains will consequently not drive the course of disease as much ahead as R5 strains in early stages of disease. Therefore the model predicts that non-progressors may even have more X4 strains than R5 strains without experiencing a coreceptor switch. The intricate dependency of the coreceptor switch on the numbers and composition of strains is analysed in detail in the following section.

### The coreceptor switch

**Figure 3:**
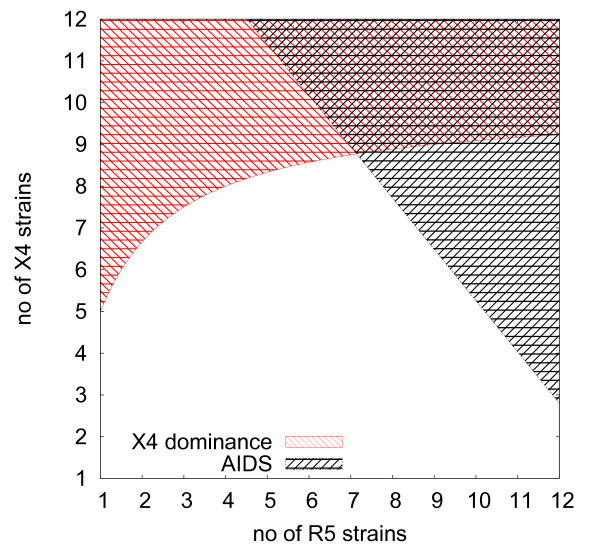
**X4 dominance and the onset of AIDS**. The figure shows how the dominance of X4 viral load and the onset of AIDS depends on the number of X4 and R5 strains present in the system according to equations (3) and (7), for parameters cf. table 2.

The coreceptor switch is a phenomenological observation for which a corresponding process has to be characterised in our model. As the simulations start with a dominance of R5 viruses due to the bias in immune recognition (i.e. *p*^*X*4 ^> *p*^*R*5^) we define that a coreceptor switch has occurred if the viral load is dominated by X4 viruses at the onset of AIDS which is determined in the model by either exceeding a viral load *v*_*max *_or the critical numbers of strains *n*^*R*5 ^and *n*^*X*4 ^(cf. Fig. 2). From

it can be seen that it depends on the number and composition of viral strains in the system which type of virus dominates, i.e. when the trade-off between replication rate and sensitivity to the immune response favours X4 viruses. X4 viral load dominates in a patient who has not yet reached the terminal stage of disease if

which corresponds to the area shaded in red in Fig. 3. In early infection X4 strains do not impose a high viral load and the number of X4 strains required to dominate the viral population in the presence of R5 viruses is usually not found in a patient. This picture changes in late infection where a smaller fraction of X4 strains among all strains can be sufficient for dominance in in viral load. This can be seen in Fig. 3 which shows graphically what strain composition leads to a dominance in X4 viral load (red shaded area) and when the system is destabilised indicating the onset of AIDS (grey shaded area). The saturation behaviour of equation (7) implies that dominance of X4 viruses in viral load can be attained with a minority of X4 strains as soon as

Note that this situation is only observed in the latent phase defined by equation (3) if X4 viruses gain sufficiently fast replicative fitness, i.e. .

**Figure 4:**
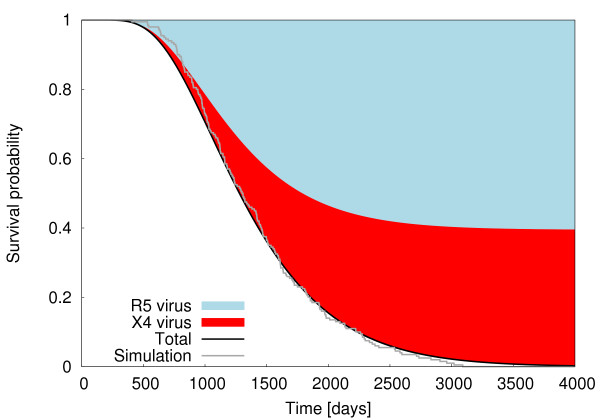
**Survival distribution and the coreceptor switch**. The probability to have reached the final stage of disease (black) either with dominance in viral load of R5 viruses (blue) or X4 viruses (red) according to the master equation (5), the survival distribution sampled from 200 simulations of equation (1) (grey) agree well with the master equation's predictions i.e. giving support to the assumption of quasi-stationary viral populations, parameters according to table 2.

Within the model the occurrence of a coreceptor switch depends on the probabilistically chosen evolutionary path in the patient (cf. Fig. 2) - i.e. whether a path is chosen that leads directly to destabilisation and AIDS or whether the final stage is reached after a switch in coreceptor usage. The probabilities for either choice are shown in Fig. 4. It shows that for any newly infected patient the probabilities of eventually reaching the final stage of disease with dominance of R5 viruses or X4 viruses are of the same order of magnitude with the parameter set chosen in in table 2. Dominance of X4 viruses is only possible with a certain amount of strains and immune activation being established and is therefore associated with an increased number of strains and a progressed stage of disease.

The results from the probabilistic approach considering equilibrium viral load according to equation (5) are compared with survival curves sampled from simulations of equations (1) based on the actual viral load. The good agreement shows that the flow of mutants is strongly determined by the equilibrium viral load (cf. Additional file 2 and 3, Figure S2 and S3).

As the average time to the onset of AIDS as well as the mean residence times in a given stage decrease with the accumulation of mutant strains (cf. equation (16)) patients with dominance in X4 viral load have a worsened prognosis in this respect. The higher burden in viral load associated with the higher number of strains leads to an accelerated progression of disease (cf. equation (16) and Figs. 2 and 3).

Note that suppression of R5 viruses as facilitated by coreceptor blockers [12,21-23] does not only reduce R5 viral load in the model but also diminishes the X4 viral load due to reduced immune activation (cf. Additional file 4, Figure S4).

## Conclusion

A mechanistic assessment of the processes leading to the coreceptor switch in HIV infected patients is difficult - and maybe even inadequate - due to the large variability and stochasticity of the underlying processes *in vivo*. Quantities such as viral growth or clearance rates are often only known in very specific settings without detailed knowledge of possible hidden dependencies from the underlying dynamical processes - and can in consequence point in contradictory directions. The observation that certain HIV strains may be present in a patient for a long time before they grow to a detectable viral load [39,40] hints however towards a picture of a dynamical change in the environmental conditions in the host. This holds specifically for X4 viruses [40].

The current model provides a coarse grained, probabilistic picture of possible dynamical scenarios of the coreceptor switch without going yet into the details of virus replication and immune response. This allows to study the basic dynamical features and conditions for a coreceptor switch. In the model, the viral quasispecies [41] is generated by a probabilistic process under the pressure of the immune system. Its composition depends on the randomly chosen evolutionary path that may show some biases due to environmental conditions. These include the viral replication efficiencies based on adequate target cells as well as constraints of immune recognition or therapy. The answer to the question whether a coreceptor switch has occurred is determined by the composition of the viral quasispecies found at the time when the host's immune system loses control and the viral load diverges, i.e. at the onset of AIDS in the model system. One prediction is that the suppression of R5 viruses in the host will not necessarily result in an expansion of X4 viruses. The control of R5 viruses can even delay or prevent the emergence of considerable X4 viral load - which strongly depends on the constitution of the host, i.e. in biases in the choice of the possible evolutionary paths. This is well in accordance with the studies done for a newly licensed drug which blocks the CCR5 receptor [22,23,40]. The model provides a stochastic framework to investigate R5 and X4 virus evolution in a dynamic environment, however staying with a qualitative description of the cellular processes. This is a strong simplification of the complex interaction network that interlinks HIV and the hosts immune system. The growing knowledge about their interactions [15,27,32,42] will be implemented within a refined model. This should combine the advantages of this stochastic approach with more details on the involved immune cells populations - both with regard to their role in the immune response and their contribution to persistent immune activation and viral replication - to allow for more quantitative predictions.

## Methods

### Virus evolution

The differential equation for the evolution of viral load and immune response (1) have been solved numerically with routine rk4 from the Math::RungeKutta module for PERL. The system is initialised with one R5 viral strain and one X4 viral strain with *v*^*R*5^(0) = *v*^*X*4^(0) = 1*V L*, i.e. one unit of viral load for each subtype. During each integration step of duration *dt *= 0.02*d *a new mutant arises with probability *dt *× *p*_*m *_× *v *= 0.02*d *× 0.01*d*^-1^*VL*^-1 ^× *v *proportional to the viral load *v*. *R*5 and *X*4 mutants occur with equal probability.

Equation (2) can be derived from equations (1) following [20] (chapt. 13) by assuming the stationary case, i.e. , i.e.

for {*v*_*i*_|*v*_*i *_≠ 0}. With the parameters from table 2 this results in

with  being the total viral load and  and  the viral load of one *R*5 or *X*4 strain respectively. The total viral load attributed to *R*5 and *X*4 viruses can be derived to

leading to equations (6) and (7) (*v*(*n*^*R*5^, *n*^*X*4^) = *v*^*R*5^(*n*^*R*5^, *n*^*X*4^) + *v*^*X*4^(*n*^*R*5^, *n*^*X*4^)). Note that *v*^*R*5^(*n*^*R*5^, *n*^*X*4^) < 0 corresponds to the case in which no R5 viruses can co-exist with X4 viruses, i.e. a total coreceptor switch [20].

In case of a reduction of the growth rate in R5 viruses from *r *to *r*_*CCR*5 _<*r *the above equations change to

leading to a condition for the onset of AIDS

that allows for more virus strains and the condition for dominance of X4 viruses becomes

Note that less R5 viruses can coexist with X4 viruses in case of *r*_*CCR*5 _<*r*, i.e. neither R5 infection nor X4 infection (due to lack of immune activation) can progress as fast as without therapy.

### Probabilistic approach

Assuming fast equilibration of viral load relative to the time scale of emergence of new mutants allows to model the evolution in the number of viral strains as a Markov process [35] with constant transmission rates between states which are proportional to the equilibrium viral load. With *P*(*n*^*R*5^, *n*^*X*4^, *t*) being the probability to find *n*^*R*5 ^*R*5 strains and *n*^*X*4 ^*X*4 strains at time *t *the master equation [35] for this process can be written as

with *p*_1 _being the probability per time and viral load to mutate within the same subtype (*R*5 → *R*5, *X*4 → *X*4) and *p*_2 _being the probability per time and viral load to mutate between subtypes (*R*5 → *X*4, *X*4 → *R*5). For the data shown it holds *p*_1 _= *p*_2 _=  = 0.005*d*^-1^*VL*^-1 ^(for *p*_1 _≠ *p*_2 _cf. Additional file 1, Figure S1). The last term in equation (15) vanishes if the critical number of stains according to equation (3) or the maximal viral load *v*_*max *_= 500*V L *is exceeded leading to an absorbing boundary. The time evolution of *P*(*n*^*R*5^, *n*^*X*4^, *t*) was solved numerically with routine rk4 from the Math::RungeKutta module for PERL with the initial condition *P*(1, 1, 0) = 1 (zero otherwise) corresponding to the initialisation with one *R*5 and one *X*4 strain.

The distribution of the waiting times to the onset of AIDS on the basis of an initial distribution in strain numbers *n*^*R*5^, *n*^*X*4^can be described by a phase type distribution [36]. With *p*_1 _= *p*_2 _= , a patient's average residence time in a configuration with *n*^*R*5 ^R5 virus strains and *n*^*X*4 ^X4 virus strains is  i.e. decreasing with a growing number of viral strains and in consequence viral load *v*(*n*^*R*5^, *n*^*X*4^). This allows to derive the mean time to the onset of AIDS in a patient with *n*^*R*5 ^R5 virus strains and *n*^*X*4 ^X4 virus strains to

The mean waiting time until the onset of AIDS is derived in equation (16) by averaging the waiting times arising along all possible evolutionary paths starting form a configuration with *n*^*R*5 ^R5 virus strains and *n*^*X*4 ^X4 virus strains (cf. Additional file 5, Figure S5).

## Authors' contributions

CK carried out the study and wrote the manuscript.

## Supplementary Material

Additional file 1**Mutation between R5 and X4 viruses**. The Figure shows a comparison between the situation of homogeneous and heterogeneous mutation rates among R5 and X4 viruses. While the former case (left panel) corresponds to the situation discussed in the main paper, i.e. mutation rates being identical among and between subtypes *p*_1 _= *p*_2 _= , the latter case (right panel) has the same total mutation rate *p*_*m *_= 0.01*d*^-1 ^*V L*^-1 ^but a threefold higher probability for intra-subtype mutation than inter-subtype mutation, i.e. *p*_1 _= , *p*_2 _= . The top row shows how this assumption shifts evolutionary paths towards routes with a higher fraction of *R*5 viruses. In consequence, X4 dominance is less often attained before the onset of AIDS leading to a lower fraction of coreceptor switches. This might however be shifted to the observed levels by a stronger coupling of X4 growth to immune activation. The survival curves decay slightly steeper in the case of heterogeneous mutation patterns because more viable R5 mutants (than not yet adapted X4 mutants) are generated in the earlier stages of disease. The exact mutation rates among R5 and X4 viruses are hard to estimate, but their sequence similarity suggests them to be of a similar order of magnitude (*p*_1 _≈ *p*_2_) [33].Click here for file

Additional file 2 **Equilibrium viral load**. Viral load of R5 viruses (blue) and X4 viruses (red) in a simulation of the model described by equations (1) as shown in Fig. 1 (parameters as in table 2). In addition the equilibrium viral load according to equations (11) is shown with good agreement with the simulation data. Deviations occur only at the breakdown of the system where no equilibration can be expected any more. Click here for file

Additional file 3 **Variation of mutation rate *p*_*m*_**. Survival distribution for *p*_*m *_= 0.01 (cf. Fig. 4) and *p*_*m *_= 0.1 sampled from equations (1) (200 runs each) in comparison with the probabilistic approach (5), other parameters as in table 2. The ten-fold increase of mutation rate shrinks the time axis of the survival distribution by a factor of 10. The survival curves for *p*_*m *_= 0.1 are still close but the sampled curve decays faster than predicted by the master equation approach as mutants are already likely to be established in the initial viral peak, i.e. do not allow for equilibration. Click here for file

Additional file 4 **Suppression of R5 viruses and viral load**. The density plots show the equilibrium viral load determined by equations (11,12) (as visualised in Additional file 2, Figure S2) depending on the numbers of R5 and X4 virus strains being present. The top row shows the equilibrium viral load for R5 viruses *v*^*R*5^(*n*^*R*5^, *n*^*X*5^) and X5 viruses *v*^*X*4^(*n*^*R*5^, *n*^*X*4^) for parameter values as depicted in Table 2. The bottom row shows the same situation, however, with the growth rate of R5 viruses halved to 1*d *^-1 ^leading to a situation in which hardly any R5 viral load can be established. This however, results at the same time in a lower viral load from X4 viruses (cf. right panel). In consequence, the model predicts an indirect positive effect from the suppression by R5 viruses as induced by CCR5 blockers. Click here for file

Additional file 5**Mean waiting time  to the onset of AIDS**. The Figure shows a graphical representation of equation (16) in the *n*^*R*5^-*n*^*X*4^-plane showing the probability to find *n*^*R*5 ^R5 strains and *n*^*X*4 ^X4 strains in a patient at 1200 days since infection (cf. Fig. 2). The mean waiting time to the onset of AIDS  is determined by averaging the waiting times along all possible evolutionary paths. Therefore,  decreases with a growing number of strains (here: *T*_4,4 _> *T*_6,5_).Click here for file
