# Supplementary figures and images for: Understanding the HIV coreceptor switch from a dynamical perspective
Source: BMC Evol Biol. 2009 Nov 30;9:274. doi: 10.1186/1471-2148-9-274 (PMC2797020; doi:10.1186/1471-2148-9-274)

# homogeneous mutations

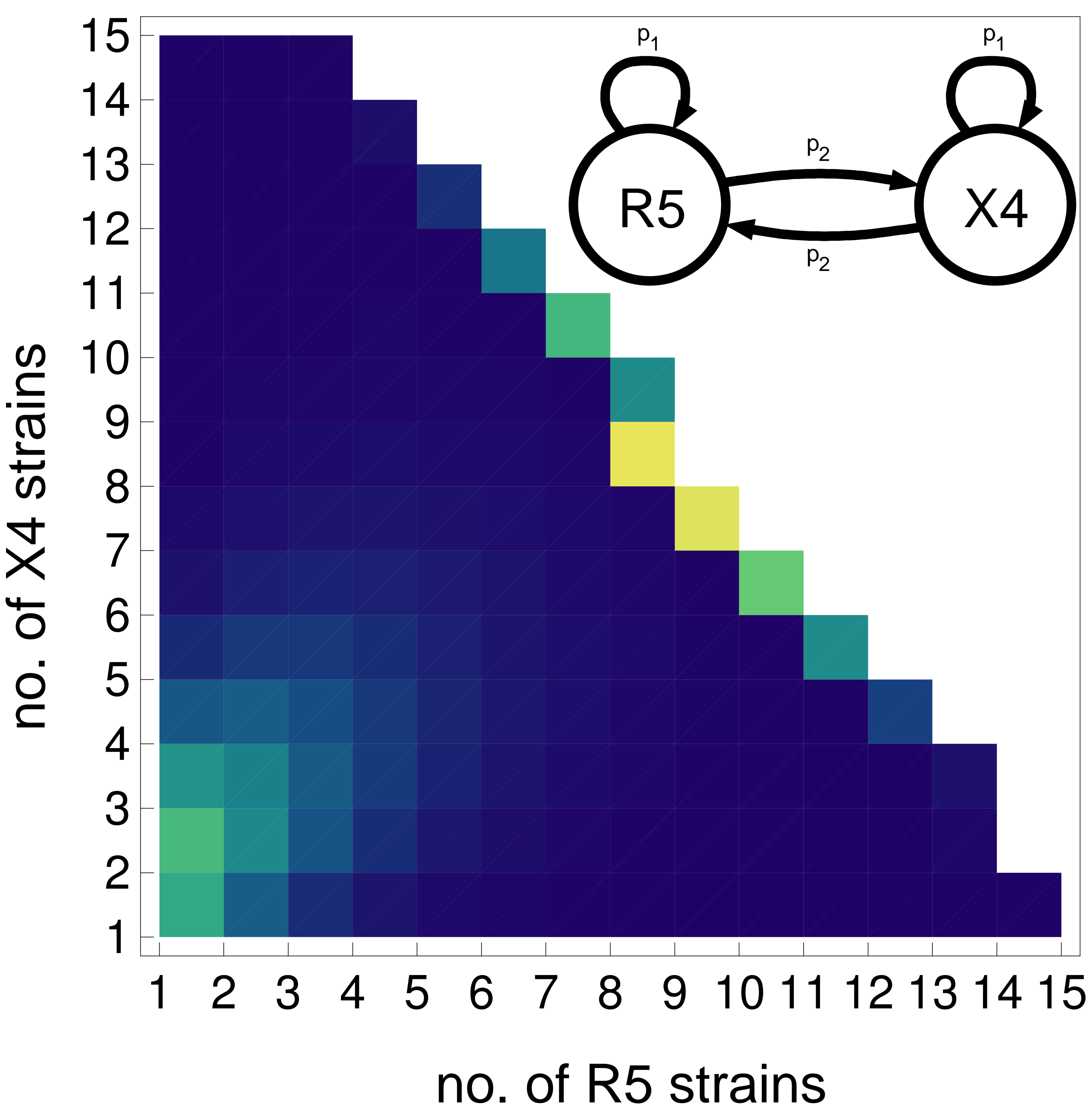

# heterogeneous mutations

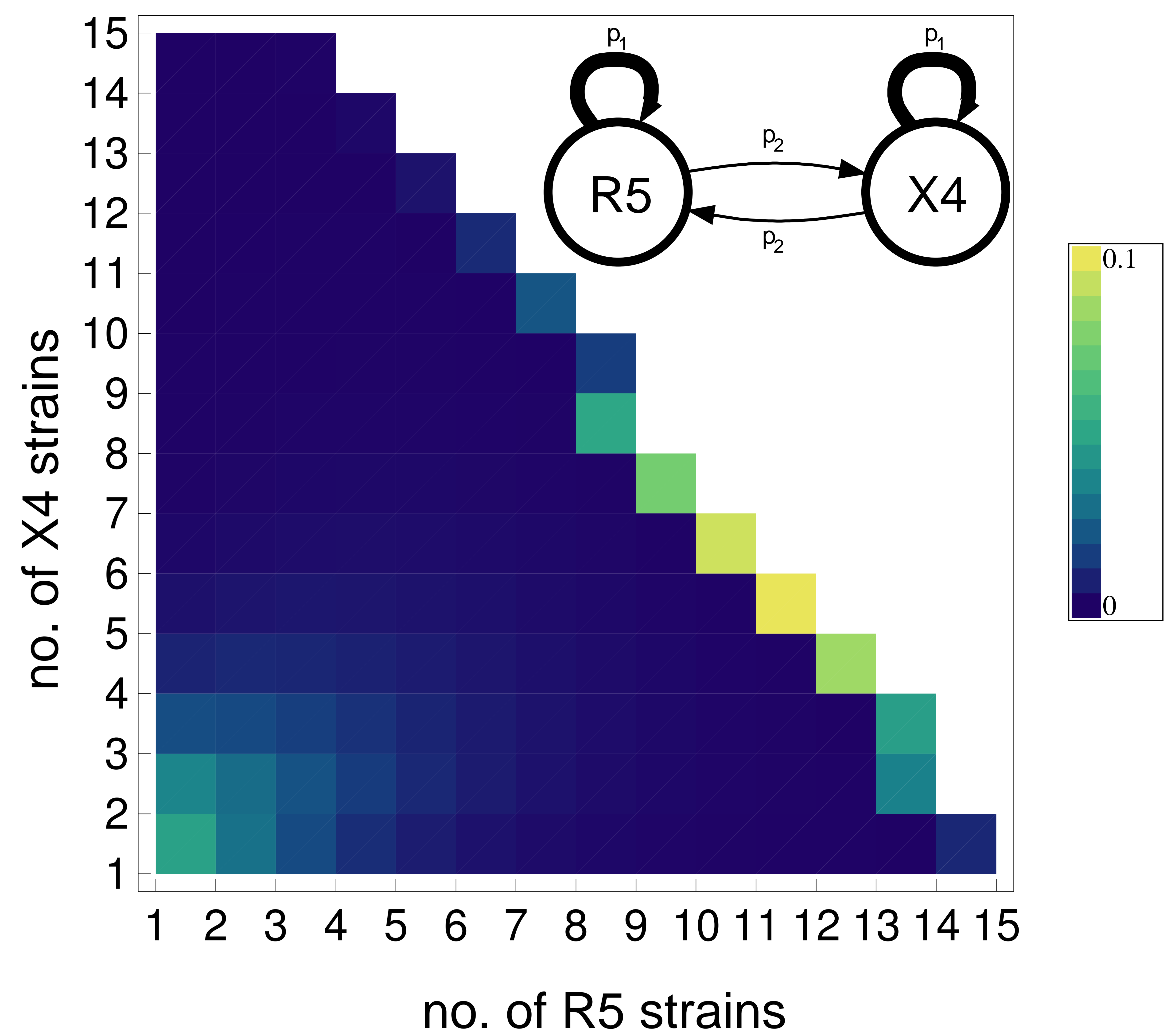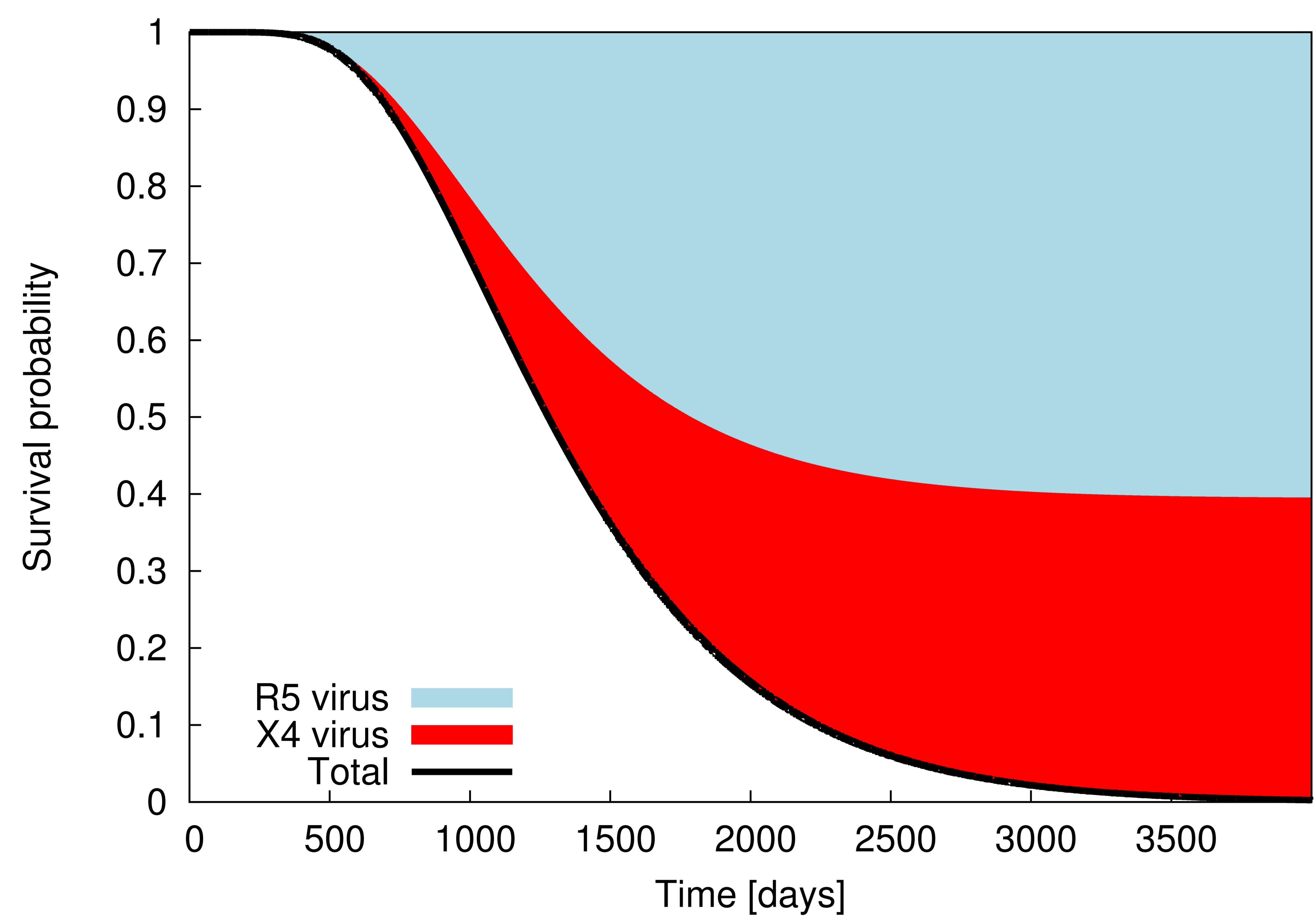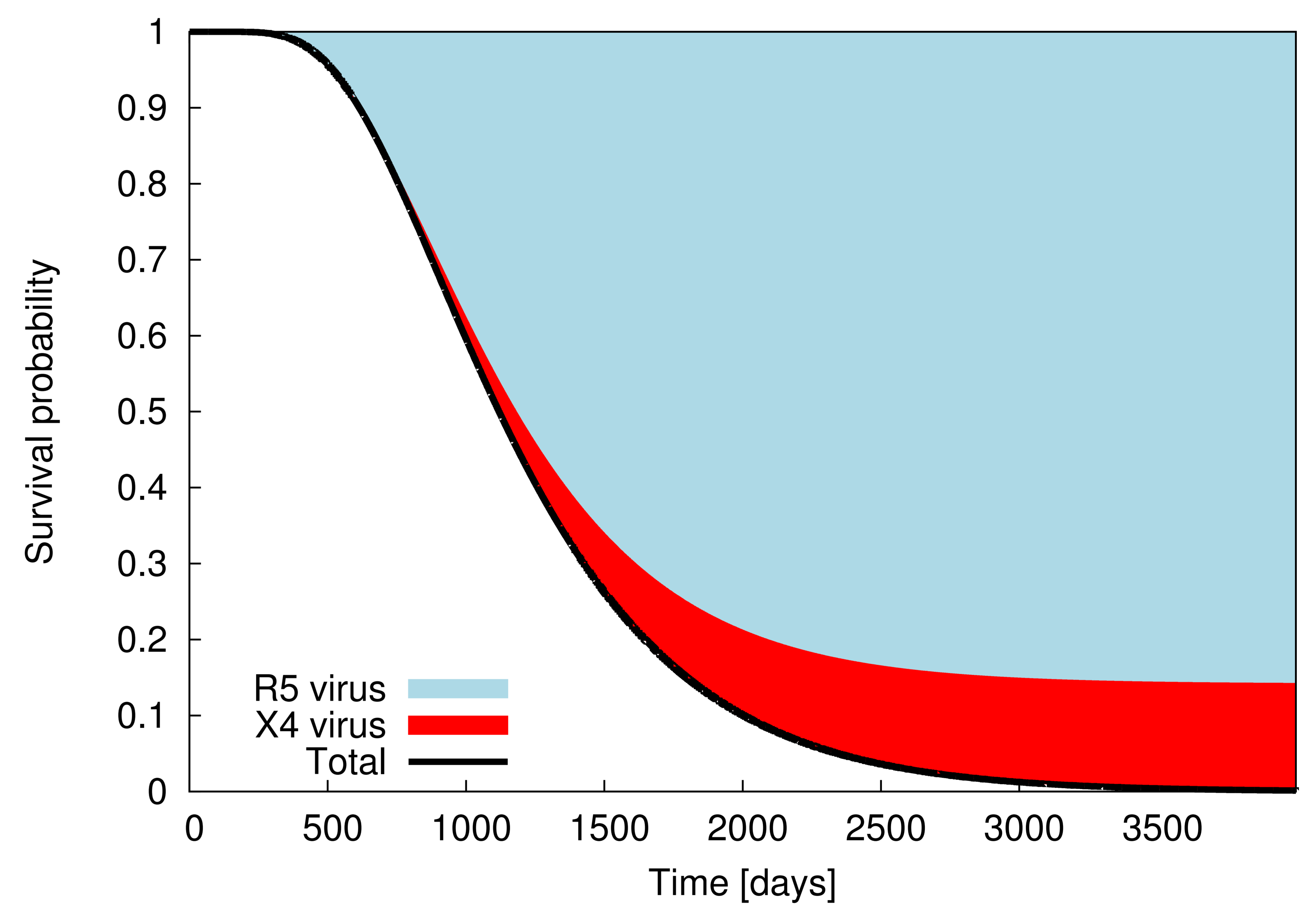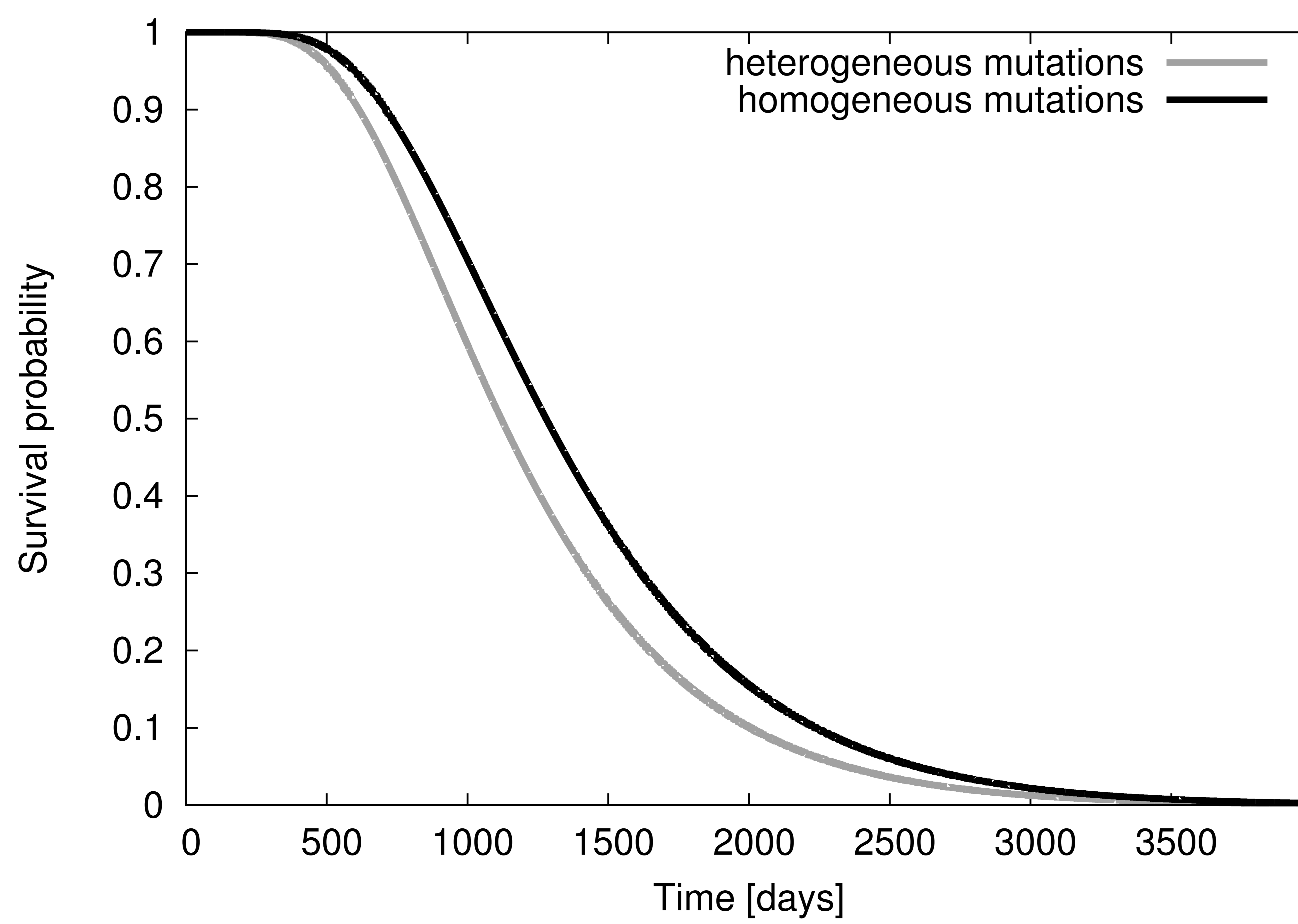

Supplement: Additional file 1 — Mutation between R5 and X4 viruses. The Figure shows a comparison between the situation of homogeneous and heterogeneous mutation rates among R5 and X4 viruses. While the former case (left panel) corresponds to the situation discussed in the main paper, i.e. mutation rates being identical among and between subtypes p1 = p2 = , the latter case (right panel) has the same total mutation rate pm = 0.01d-1 V L-1 but a threefold higher probability for intra-subtype mutation than inter-subtype mutation, i.e. p1 = , p2 = . The top row shows how this assumption shifts evolutionary paths towards routes with a higher fraction of R5 viruses. In consequence, X4 dominance is less often attained before the onset of AIDS leading to a lower fraction of coreceptor switches. This might however be shifted to the observed levels by a stronger coupling of X4 growth to immune activation. The survival curves decay slightly steeper in the case of heterogeneous mutation patterns because more viable R5 mutants (than not yet adapted X4 mutants) are generated in the earlier stages of disease. The exact mutation rates among R5 and X4 viruses are hard to estimate, but their sequence similarity suggests them to be of a similar order of magnitude (p1 ≈ p2) [33]. [file 1471-2148-9-274-S1.PDF]

viral load

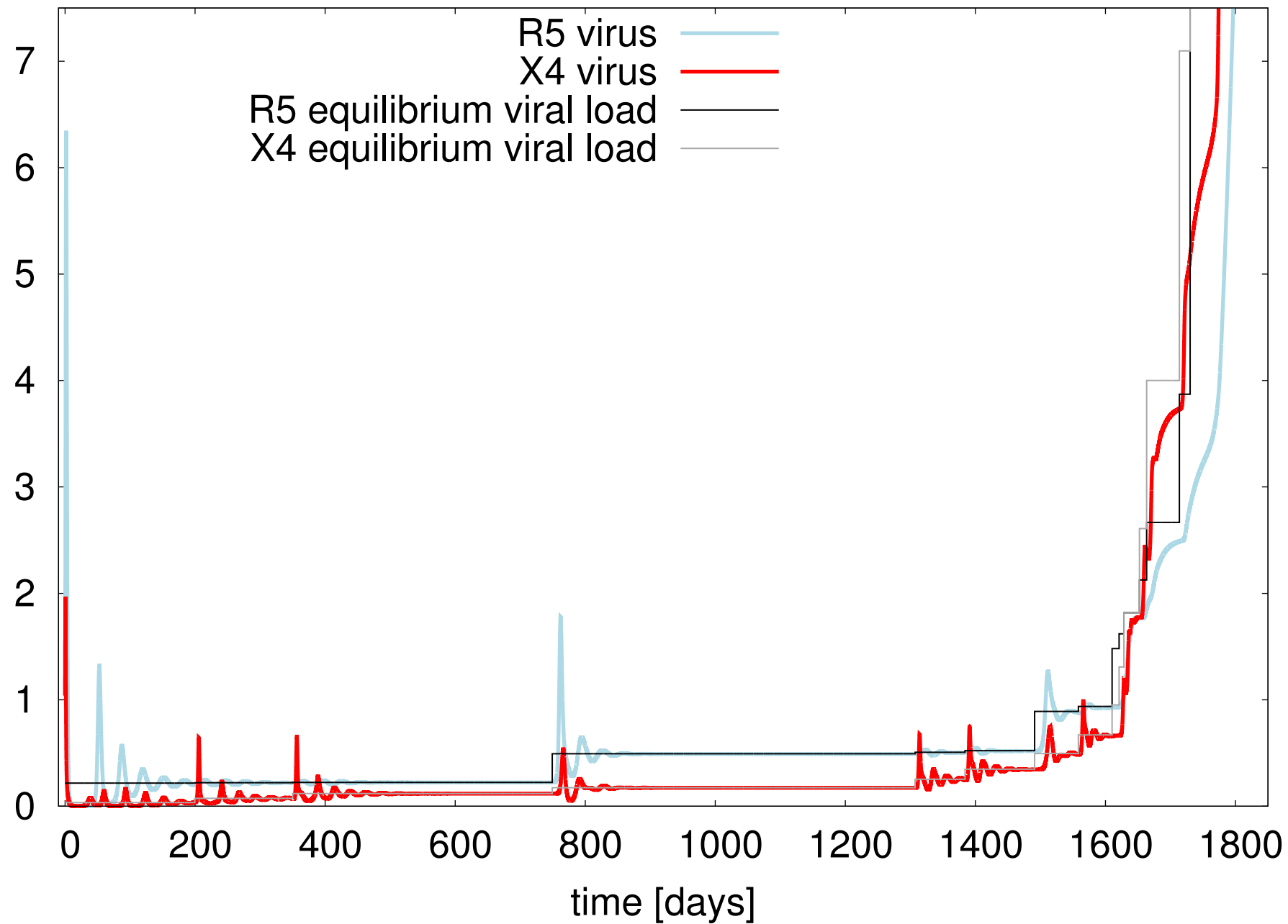

Supplement: Additional file 2 — Equilibrium viral load. Viral load of R5 viruses (blue) and X4 viruses (red) in a simulation of the model described by equations (1) as shown in Fig. 1 (parameters as in table 2). In addition the equilibrium viral load according to equations (11) is shown with good agreement with the simulation data. Deviations occur only at the breakdown of the system where no equilibration can be expected any more. [file 1471-2148-9-274-S2.PDF]

Survival probability

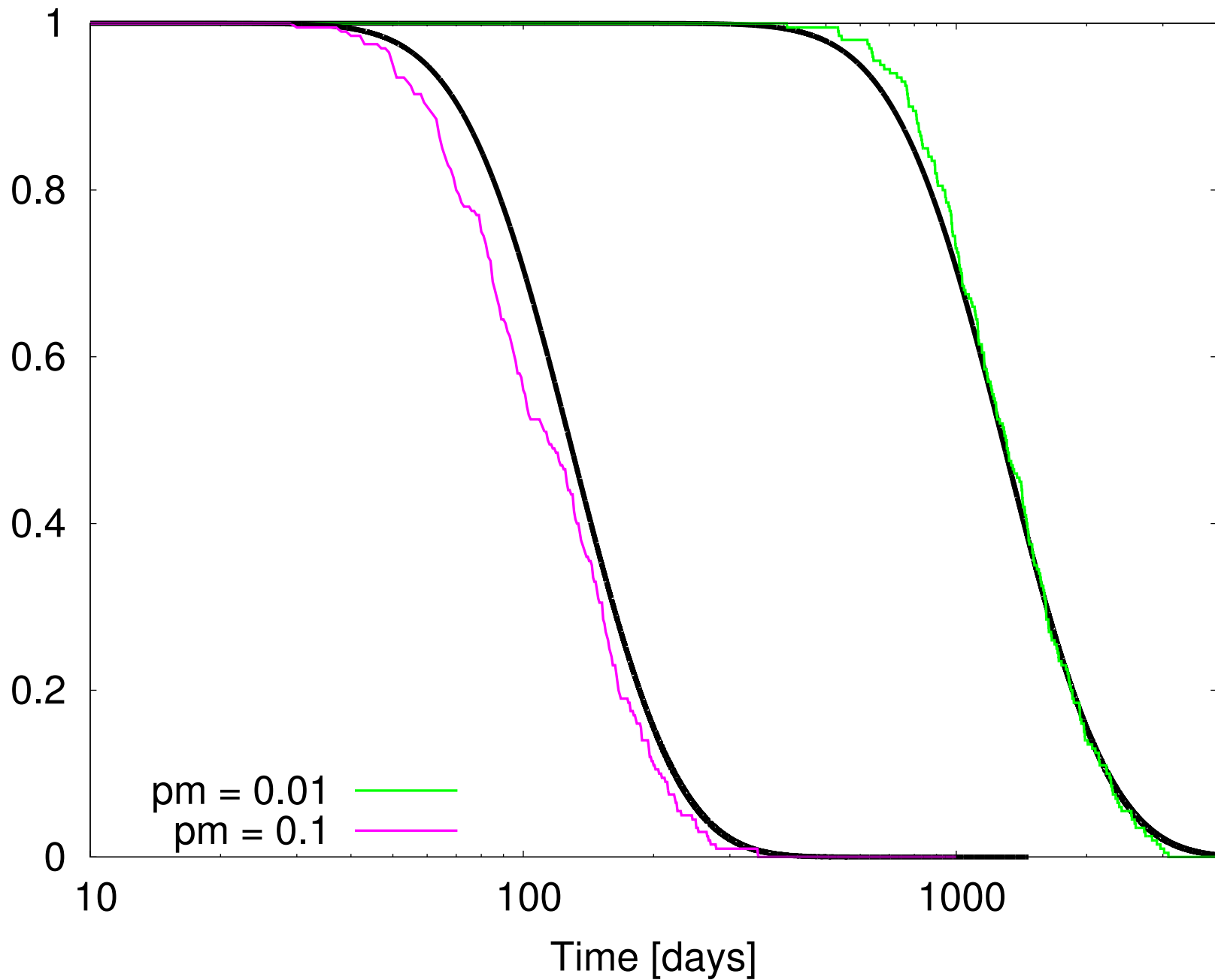

Supplement: Additional file 3 — Variation of mutation rate pm. Survival distribution for pm = 0.01 (cf. Fig. 4) and pm = 0.1 sampled from equations (1) (200 runs each) in comparison with the probabilistic approach (5), other parameters as in table 2. The ten-fold increase of mutation rate shrinks the time axis of the survival distribution by a factor of 10. The survival curves for pm = 0.1 are still close but the sampled curve decays faster than predicted by the master equation approach as mutants are already likely to be established in the initial viral peak, i.e. do not allow for equilibration. [file 1471-2148-9-274-S3.PDF]

no. of X4 strains

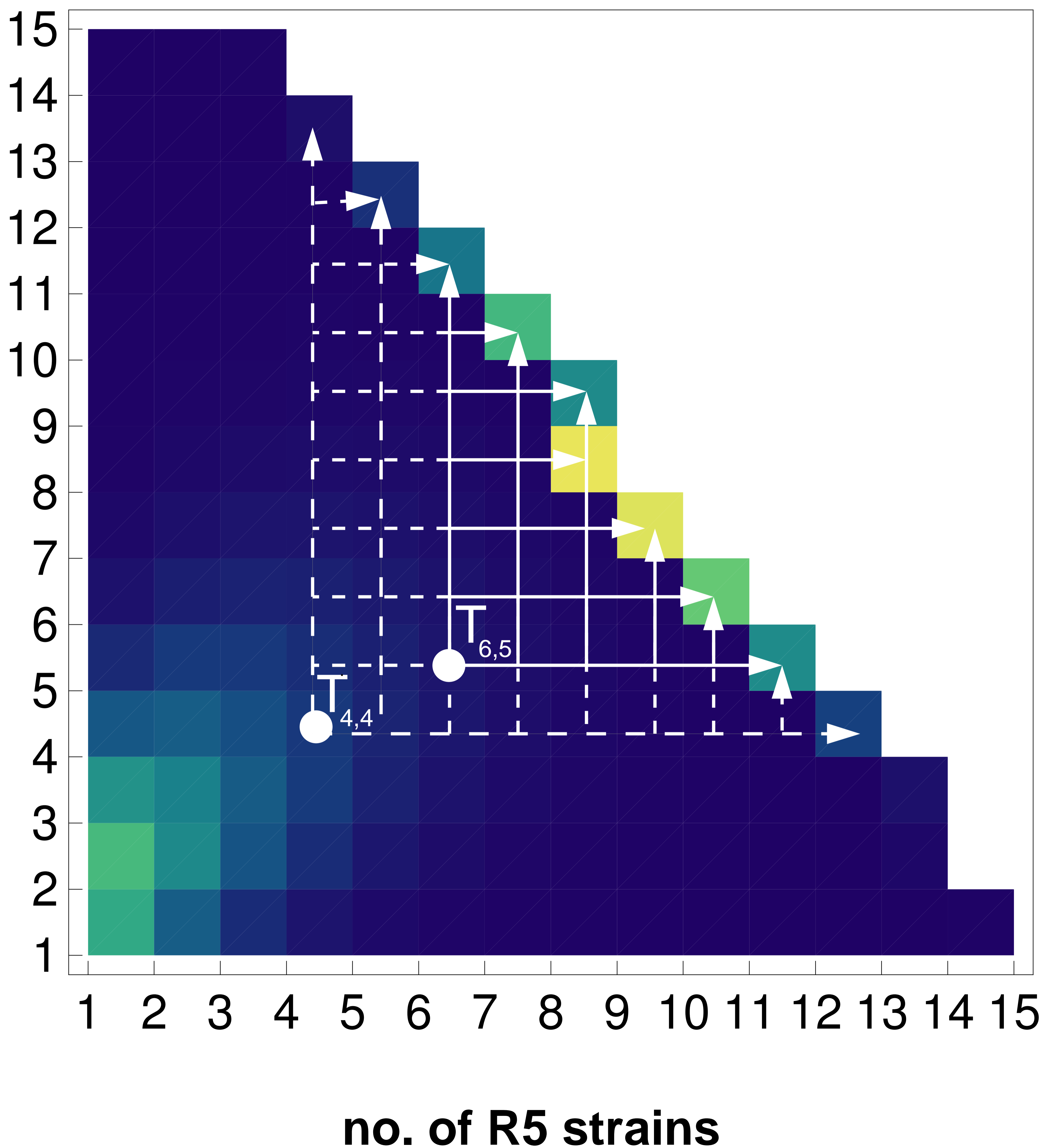

0.1

0

no. of R5 strains

Supplement: Additional file 5 — Mean waiting time to the onset of AIDS. The Figure shows a graphical representation of equation (16) in the nR5-nX4-plane showing the probability to find nR5 R5 strains and nX4 X4 strains in a patient at 1200 days since infection (cf. Fig. 2). The mean waiting time to the onset of AIDS is determined by averaging the waiting times along all possible evolutionary paths. Therefore, decreases with a growing number of strains (here: T4,4 > T6,5). [file 1471-2148-9-274-S5.PDF]
