# Supplementary material for: Understanding the HIV coreceptor switch from a dynamical perspective
Source: BMC Evol Biol. 2009 Nov 30;9:274. doi: 10.1186/1471-2148-9-274 (PMC2797020; doi:10.1186/1471-2148-9-274)
Supplement: Additional file 4 — Suppression of R5 viruses and viral load. The density plots show the equilibrium viral load determined by equations (11,12) (as visualised in Additional file 2, Figure S2) depending on the numbers of R5 and X4 virus strains being present. The top row shows the equilibrium viral load for R5 viruses vR5(nR5, nX5) and X5 viruses vX4(nR5, nX4) for parameter values as depicted in Table 2. The bottom row shows the same situation, however, with the growth rate of R5 viruses halved to 1d -1 leading to a situation in which hardly any R5 viral load can be established. This however, results at the same time in a lower viral load from X4 viruses (cf. right panel). In consequence, the model predicts an indirect positive effect from the suppression by R5 viruses as induced by CCR5 blockers. [file 1471-2148-9-274-S4.PDF]

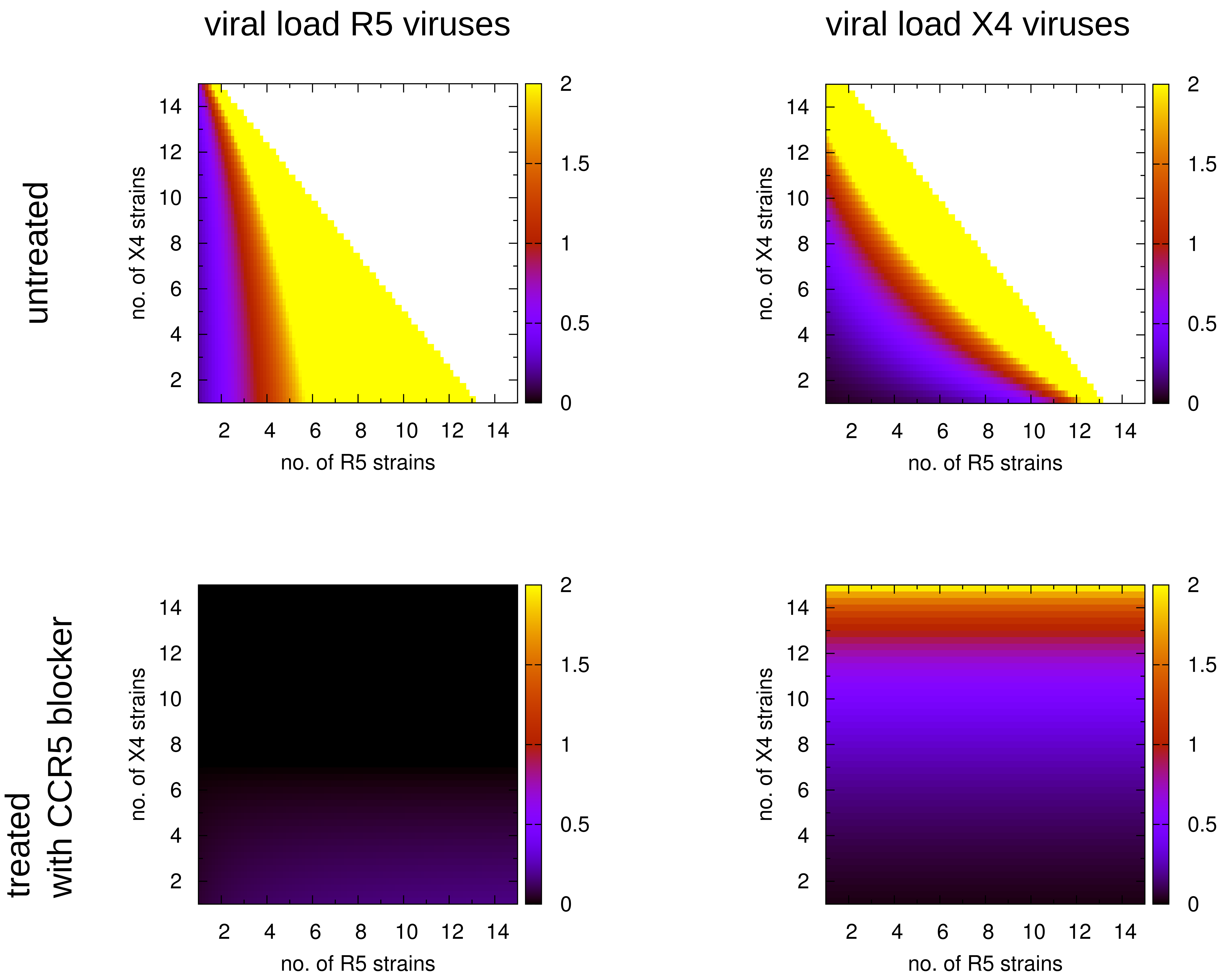
viral load R5 viruses
viral load X4 viruses
untreated
treated
with CCR5 blocker
no. of X4 strains
no. of R5 strains
2
4
6
8
10
12
14
0
0.5
1
1.5
2
